# Hispidin induces autophagic and necrotic death in SGC-7901 gastric cancer cells through lysosomal membrane permeabilization by inhibiting tubulin polymerization

**DOI:** 10.18632/oncotarget.15935

**Published:** 2017-03-06

**Authors:** Long-Xian Lv, Zhen-Xing Zhou, Zhan Zhou, Li-Jiang Zhang, Ren Yan, Zhao Zhao, Li-Ya Yang, Xiao-Yuan Bian, Hui-Yong Jiang, Yu-Dong Li, Yi-Sheng Sun, Qin-Qin Xu, Gui-Li Hu, Wen-Jun Guan, Yong-Quan Li

**Affiliations:** ^1^ Institute of Pharmaceutical Biotechnology and College of Pharmaceutical Sciences, Zhejiang University, 310058 Hangzhou, China; ^2^ State Key Laboratory for Diagnosis and Treatment of Infectious Diseases, Collaborative Innovation Center for Diagnosis and Treatment of Infectious Diseases, The First Affiliated Hospital, College of Medicine, Zhejiang University, 310003 Hangzhou, China; ^3^ Department of Basic Medicine, College of Medicine, Zhejiang University, 310058 Hangzhou, China

**Keywords:** hispidin, autophagy, necrotic cell death, lysosomal membrane permeabilization, microtubule depolymerization

## Abstract

Hispidin and its derivatives are widely distributed in edible mushrooms. Hispidin is more cytotoxic to A549, SCL-1, Bel7402 and Capan-1 cancer cells than to MRC5 normal cells; by contrast, hispidin protects H9c2 cardiomyoblast cells from hydrogen peroxide-induced or doxorubicin-induced apoptosis. Consequently, further research on how hispidin affects normal and cancer cells may help treat cancer and reduce chemotherapy-induced side effects. This study showed that hispidin caused caspase-independent death in SGC-7901 cancer cells but not in GES-1 normal cells. Hispidin-induced increases in LC3-II occurred in SGC-7901 cells in a time independent manner. Cell death can be partially inhibited by treatment with *ATG5* siRNA but not by autophagy or necroptosis inhibitors. Ultrastructural evidence indicated that hispidin-induced necrotic cell death involved autophagy. Hispidin-induced lysosomal membrane permeabilization (LMP) related to complex cell death occurred more drastically in SGC-7901 cells than in GES-1 cells. Ca^2+^ rather than cathepsins from LMP contributed more to cell death. Hispidin induced microtubule depolymerization, which can cause LMP, more drastically in SGC-7901 cells than in GES-1 cells. At 4.1 μM, hispidin promoted cell-free tubulin polymerization but at concentrations higher than 41 μM, hispidin inhibited polymerization. Hispidin did not bind to tubulin. Alterations in microtubule regulatory proteins, such as stathmin phosphorylation at Ser^16^, contributed to hispidin-induced SGC-7901 cell death. In conclusion, hispidin at concentrations higher than 41 μM may inhibit tubulin polymerization by modulating microtubule regulatory proteins, such as stathmin, causing LMP and complex SGC-7901 cell death. This mechanism suggests a promising novel treatment for human cancer.

## INTRODUCTION

Recently, the number of new cases of gastric cancer has grown to more than 900,000 each year worldwide [1]. Approximately 70% of these patients die within 5 years of diagnosis. Gastric cancer has become the third most lethal cancer worldwide, the second in Asia, and the first in China [1, 2]. Epidemiological investigations have revealed that environmental factors, particularly diet and *Helicobacter pylori* infection, play important roles in the generation and development of gastric cancer.

Hispidin (6-(3, 4-dihydroxystyryl)-4-hydroxy-2-pyrone, calculated relative molecular mass 246.2) and its derivatives are widely distributed in edible mushrooms such as *Phellinus linteus* [3–6]. On one hand, the administration of three successive doses of hispidin (between 0.1 μM and 1 μM) on three successive days led to a 100-fold increase in cytotoxic activity against the A549 human lung cancer cell line, SCL-1 squamous cancer cell line, Bel7402 liver cancer cell line and Capan-1 pancreatic cancer cell line compared to the normal pulmonary cell line MRC5 (50%) [7]. When administered at doses greater than 406 μM, hispidin significantly induced ROS-mediated apoptosis of CMT-93 and HCT 116 cancer cells over 24 h, although its effects on the corresponding normal cells were not shown [8]. On the other hand, hispidin was found to protect H9c2 cardiomyoblast cells against hydrogen peroxide-induced apoptosis by reducing intracellular ROS production and activating the Akt/GSK-3β and ERK1/2 signaling pathways [9]. Hispidin treatment decreased the doxorubicin-induced activation of caspase 9 and p66Shc alterations in H9c2 cardiomyoblast cells, thus providing a promising therapeutic approach for circumventing doxorubicin-induced cardiotoxicity [10]. Since hispidin seemed to play different roles under these conditions mentioned above, further research on how hispidin affects normal and cancer cells may help to treat cancer and to prevent chemotherapy-induced side effects. In this study, we investigated the different effect of hispidin on the human gastric cancer cell line SGC-7901 and the immortalized human gastric epithelial cell line GES-1 to illustrate the mechanism by which hispidin induces more cytotoxicity in tumor cells.

## RESULTS

### Hispidin induces caspase-independent cell death in SGC-7901 cells

**Figure 1:**
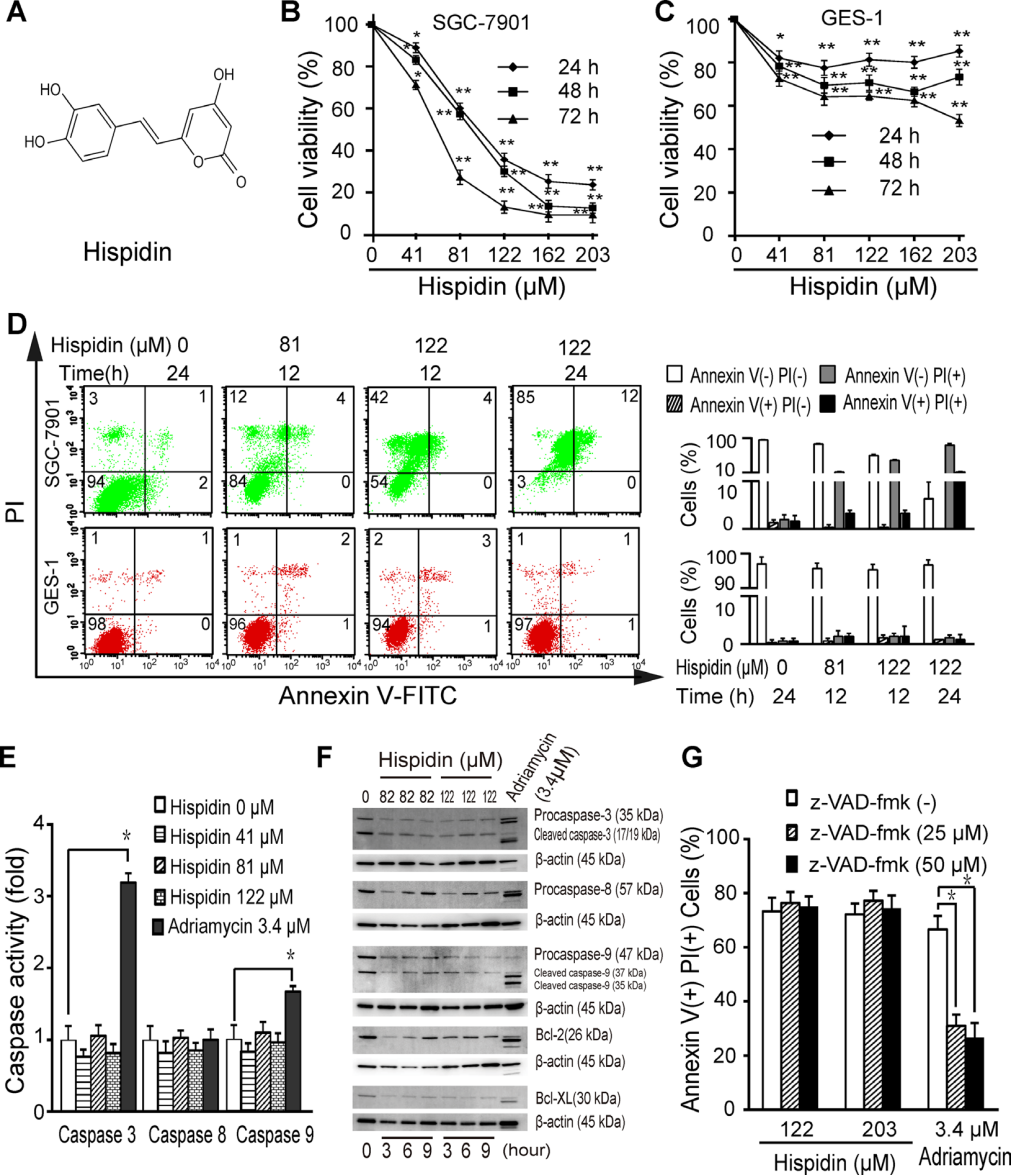
Hispidin induces caspase-independent cell death in SGC-7901 cells (**A**) Chemical structure of hispidin. Cells were incubated with hispidin (41, 82 or 122 μM) or 0.1% DMSO for 12, 24, 48 or 72 h. The viability of SGC-7901 (**B**) and GES-1 (**C**) cells was determined using the MTT assay. (**D**) Cells were incubated with hispidin or 0.1% DMSO and then were assayed for phosphatidyl serine externalization and PI permeability. (**E**) SGC-7901 cells were treated with 41, 81, or 122 μM hispidin; 0.1% DMSO; or 3.4 μM Adriamycin for 6 h. Then, caspase activity was examined. (**F**) SGC-7901 cells were treated with hispidin or Adriamycin. Then, caspase-3, caspase-8 and caspase-9 were detected by Western blotting using β-actin as an internal control. (**G**) SGC-7901 cells were preincubated in the presence or absence of 25 or 50 μM z-VAD-FMK for 2 h before being treated with 122 μM hispidin. Then, the cells were examined for PI permeability.

We first determined the effect of hispidin (Figure 1A) on SGC-7901 cells and GES-1 cells. Hispidin reduced the viability of SGC-7901 cells in a time- and concentration-dependent manner (Figure 1B), with 50% inhibition (IC50) of 61 ± 11 μM at 24 h; however, 203 μM hispidin only reduced the viability of GES-1 cells by 20% at 24 h (Figure 1C). Hispidin triggered the appearance of bright blue nuclei (Hoechst) in SGC-7901 cells, but unlike Adriamycin, it did not induce the appearance of apoptotic bodies or significantly increase the green fluorescence intensity (TUNEL) in either cell line (Supplementary Figure 1A). This indicates that hispidin may not drive cell death through apoptosis. No hypodiploid peaks were observed in the hispidin-treated SGC-7901 cells, but the number of sub-G1 cells increased to 55.2% after exposure to 122 μM hispidin for 24 h, indicating a non-apoptotic DNA profile (Supplementary Figure 1B). As show in Figure 1D, hispidin-induced death of SGC-7901 cells was characterized by a loss of plasma membrane integrity, but no significant death was observed in GES-1 cells.

Hispidin did not activate caspase-3, caspase-8 or caspase-9 in SGC-7901 cells as the apoptotic control Adriamycin did (Figure 1E, Figure 1F and Supplementary Figure 1C). The broad-spectrum caspase inhibitor z-VAD-fmk failed to prevent hispidin-induced cell death (Figure 1G).

### Hispidin induces autophagic and necrotic cell death in SGC-7901 cells

**Figure 2:**
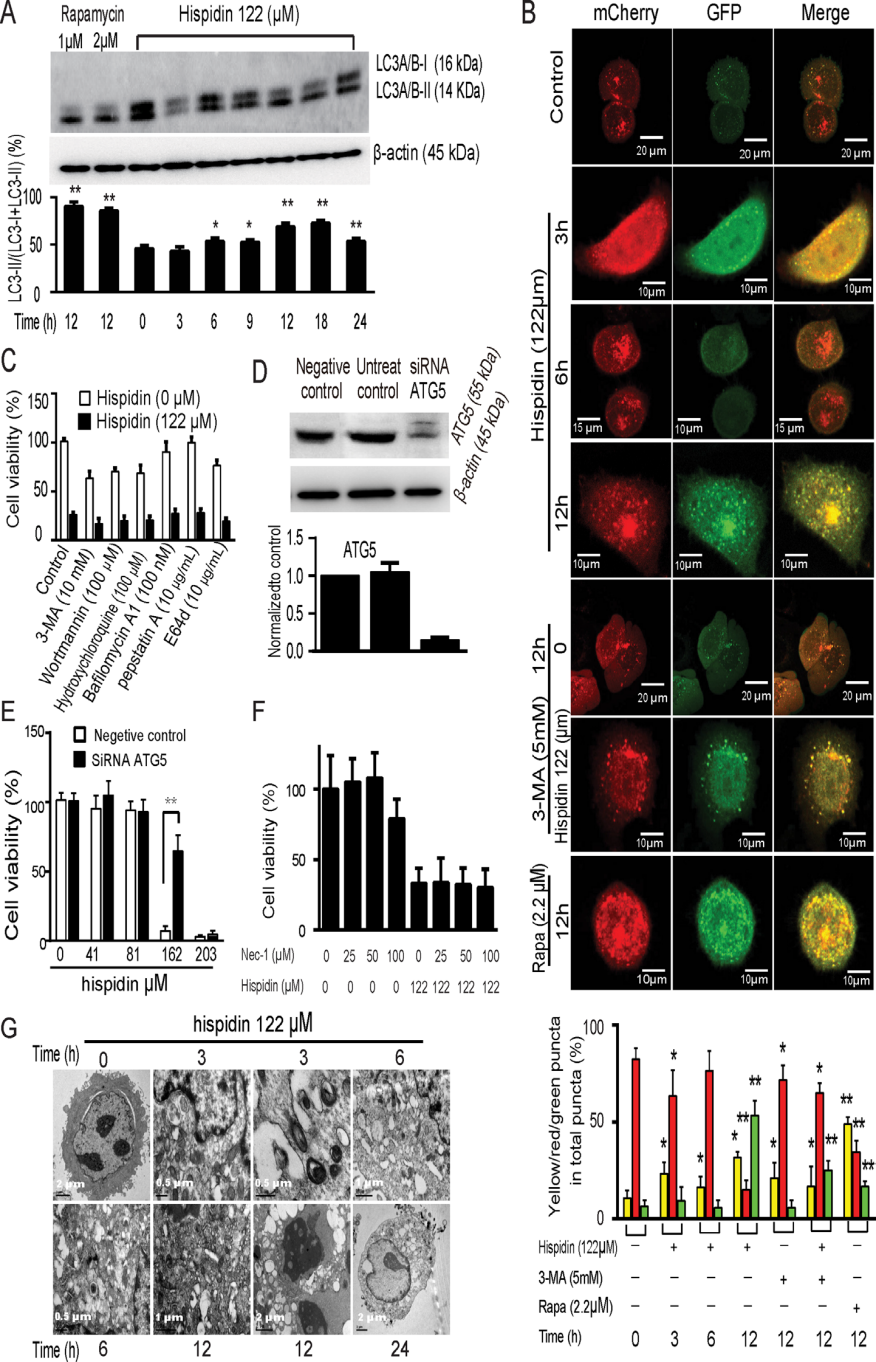
Hispidin induces necrotic cell death involving autophagy in SGC-7901 cells (**A**) SGC-7901 cells were treated with hispidin or rapamycin. Then, LC3-I and LC3-II were detected by Western blotting using β-actin as an internal control. (**B**) SGC-7901 cells were transiently transfected with mCherry-GFP-LC3B and treated with hispidin, 3-MA, or rapamycin containing medium. The colocalization of GFP and mCherry signals was analyzed. Quantitation represents the number of autophagosomes and autolysosomes per cell (*n* = 20). (**C**) After preincubation with either 10 mM 3-Methyladenine (3-MA), 100 nM wortmannin, 100 μM hydroxychloroquine, 100 nM bafilomycin A1, 10 μg/mL pepstatin A or 10 μg/mL E64d SGC-7901 cells were treated with 122 μM hispidin and assayed for cell viability. (**D** and **E**) SGC-7901 cells were transfected with scrambled RNA and *ATG5* RNA for 48 h, and ATG levels were determined by Western blot; cell viability was assayed using MTT. (**F**) SGC-7901 cells were preincubated in the presence or absence of 25, 50 or 100 nM necrostatin-1 for 2 h before being treated with 122 μM hispidin. Then, the cells were examined for viability. (**G**) Transmission electron microscopy of SGC-7901 cells. Cells were treated with 122 μM hispidin.

Next, we checked whether autophagy was activated in response to hispidin exposure. As shown in Figure 2A, hispidin-induced increases in LC3-II levels in SGC-7901 cells were observed at some points in time, but they were not time-dependent. To examine whether autophagy flux appears in hispidin-treated SGC-7901 cells, we utilized the mCherry-GFP-LC3B reporter construct (Figure 2B). Early autophagosomes display both green signal (GFP) and red signal (mCherry). Autolysosomes display only red fluorescence due to the sensitivity of GFP to the low pH in autolysosomes. Hispidin exposure caused an increase in the number of puncta and fraction of autophagosomes at 3 h, led to a drastic increase in autolysosomes at 6 h, and then induced another increase in autophagosomes in SGC-7901 cells at 12 h. This is in line with the alteration of the LC3-II level and indicates that autophagy was involved but may not be the initial cause of hispidin-induced SGC-7901 cell death. To explore the role of hispidin-induced autophagy in SGC-7901 cell death, we investigated the effects of autophagic inhibitors. As shown in Figure 2C, none of the chemical inhibitors, including 3-Methyladenine (3-MA), wortmannin, bafilomycin A1, hydroxychloroquine, E64d and pepstatin A, could inhibit hispidin-induced cell death. Then, we conducted RNA interference to knock down *ATG5*. As shown in Figure 2D and 2E, after *ATG5* was silenced, cell death was more significantly inhibited when the concentration of hispidin was lower than 122 μM. Thus, the role of autophagy may vary with the concentration of hispidin; autophagy was involved but was not the driver in SGC-7901 cell death. Necrotic cell death may also contribute to hispidin-induced cell death. To investigate whether necroptosis is involved in hispidin-induced SGC-7901 cell death, cells were pretreated with necrostatin-1 before exposure to hispidin; the results indicate that this necroptosis inhibitor did not prevent the decrease in viability of SGC-7901 cells (Figure 2F).

To further confirm the type of hispidin-induced cell death, the ultrastructure of hispidin-treated SGC-7901 cells was observed by transmission electron microscopy (Figure 2G). In the early stage, lots of vacuoles appeared; some of them touched organelles, and some had enveloped some cellular components or organelles. Subsequently, an extensive translucent cytosolic compartment, destroyed organelles, and dense bodies resembling autophagosomes were observed. After 24 h, most of the contents in the vacuoles and even the membranes of some vacuoles had disappeared. The mitochondria became rounded due to damage to their internal cisternae. The endoplasmic reticulum of some cancer cells appeared dilated. Some lipid droplets were photographed. Besides, cell membrane rupture, an important necrotic characteristic, was frequently observed. These results revealed an autophagic and necrotic cell death.

### Hispidin induces lysosomal membrane permeabilization (LMP)

**Figure 3:**
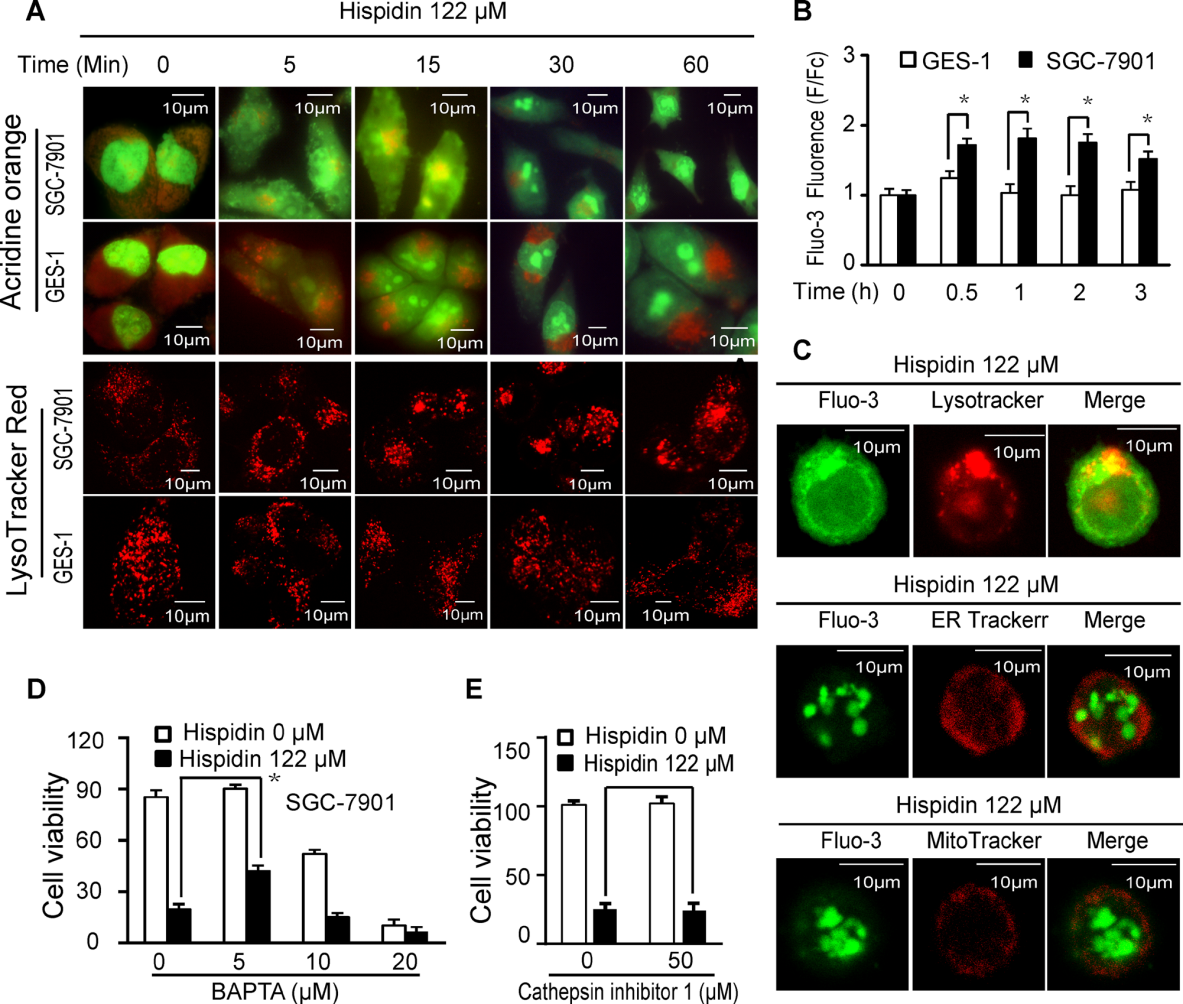
Hispidin induces lysosomal membrane permeabilization (LMP) (**A**) SGC-7901 and GES-1 cells were treated with 122 μM hispidin at different time points and then tested for LMP with acridine orange and LysoTracker red. (**B**) After being incubated with 122 μM hispidin or 0.1% DMSO for 0.5, 1, 2, or 3 h, SGC-7901 and GES-1 cells were assayed for Fluo-3 fluorescence. (**C**) SGC-7901 cells were treated with 122 μM hispidin for 30 min and examined for Fluo-3 (Ca^2+^) and the LysoTracker, ER-Tracker and MitoTracker signals. After preincubation with BAPTA AM (**D**) or cathepsin inhibitor 1 (**F**) for 2 h, SGC-7901 cells were treated with or without 122 μM hispidin for 24 h and assayed for cell viability using MTT.

Because many vacuoles appeared in hispidin-treated SGC-7901 cells, we examined whether hispidin caused LMP. To determine the reason why hispidin was more cytotoxic to SGC-7901 cells, GES-1 cells, of which no significant death was observed under the same conditions, were used as a control. A decrease in red acridine orange (AO) fluorescence (Figure 3A) and a prompt loss of the punctate LysoTracker red staining pattern (Figure 3A) were observed in SGC-7901 cells exposed to hispidin, indicating an immediate toxic effect of hispidin on lysosomes. However, in GES-1 cells, the hispidin-triggered LMP appeared milder, more controllable and less time-dependent. Remarkably, as displayed by both AO and LysoTracker red staining, aggregates of undamaged lysosomes were observed near the nucleus of hispidin-treated cells.

We subsequently examined the effects of hispidin-induced LMP on cells. First, as shown in Figure 3B, the concentration of free cytoplasmic Ca^2+^ (Fluo-3 fluorescence) in SGC-7901 cells treated with 122 μM hispidin for 0.5, 1, 2 and 3 h was 1.6-, 1.8-, 1.9- and 1.4-fold higher than that in SGC-7901 cells treated with 0.1% DMSO, respectively. However, under the same conditions, the concentration of free cytoplasmic Ca^2+^ in GES-1 cells only increased by 1.2-fold after 0.5 h, and then it slightly fluctuated by approximately 1.0-fold. When 41, 81 or 122 μM hispidin was applied for 90 min, there was a time- and concentration-dependent increase in the cytoplasmic Ca^2+^ concentration in SGC-7901 cells. However, there was nearly no increase of cytoplasmic Ca^2+^ in GES-1 cells after 1 h of treatment with 122 μM hispidin (Supplementary Figure 2A). These results showed that hispidin could more intensively increase the free cytoplasmic Ca^2+^ concentration in SGC-7901 cells. Because the cytoplasmic calcium concentration can be increased by absorption from the extracellular milieu or by release from intracellular stores such as the endoplasmic reticulum (ER), mitochondria or lysosomes, we investigated whether the hispidin-induced increase in free cytoplasmic Ca^2+^ was a result of LMP. No significant changes in hispidin-induced SGC-7901 cell death occurred when the final concentration of Ca^2+^ in the culture medium ranged from 0 to 100 μM (Supplementary Figure 2B), indicating that the increased cytoplasmic Ca^2+^ was not from the extracellular medium. Neither the inositol 1,4,5-triphosphate receptor IP3R antagonist heparin nor the ryanodine receptor antagonist procaine could prevent either the hispidin-induced cell death or the increased cytoplasmic Ca^2+^ (Supplementary Figure 2C and 2D); meanwhile, the SERCA activity increased (Supplementary Figure 2E). This suggests that the increased cytoplasmic Ca^2+^ was not from the ER. When the hispidin-treated cells were analyzed by staining, the fluo-3 fluorescence (Ca^2+^) was highly co-localized with the LysoTracker signal (Figure 3C) but was not co-localized with the ER Tracker Red signal or MitoTracker Red signal. Therefore, hispidin caused Ca^2+^ release from the lysosomes into the cytoplasm. The intracellular calcium-specific chelator BAPTA-AM (5 μM) significantly improved the viability of hispidin-treated SGC-7901 cells (Figure 3D), indicating that calcium-related signaling pathways played an important role in hispidin-induced cell death. Second, we investigated the effect of lysosomal proteases on hispidin-induced cell death. As shown in Figure 3E, the cathepsin inhibitor 1, an inhibitor of Cathepsin L, Cathepsin L2, Cathepsin S, Cathepsin K and Cathepsin B, could not reduce hispidin-induced cell death. This indicates that cathepsins were not the real driver of the process.

### Hispidin induces LMP-related nitric oxide (NO) production, contributing to SGC-7901 cancer cell death

**Figure 4:**
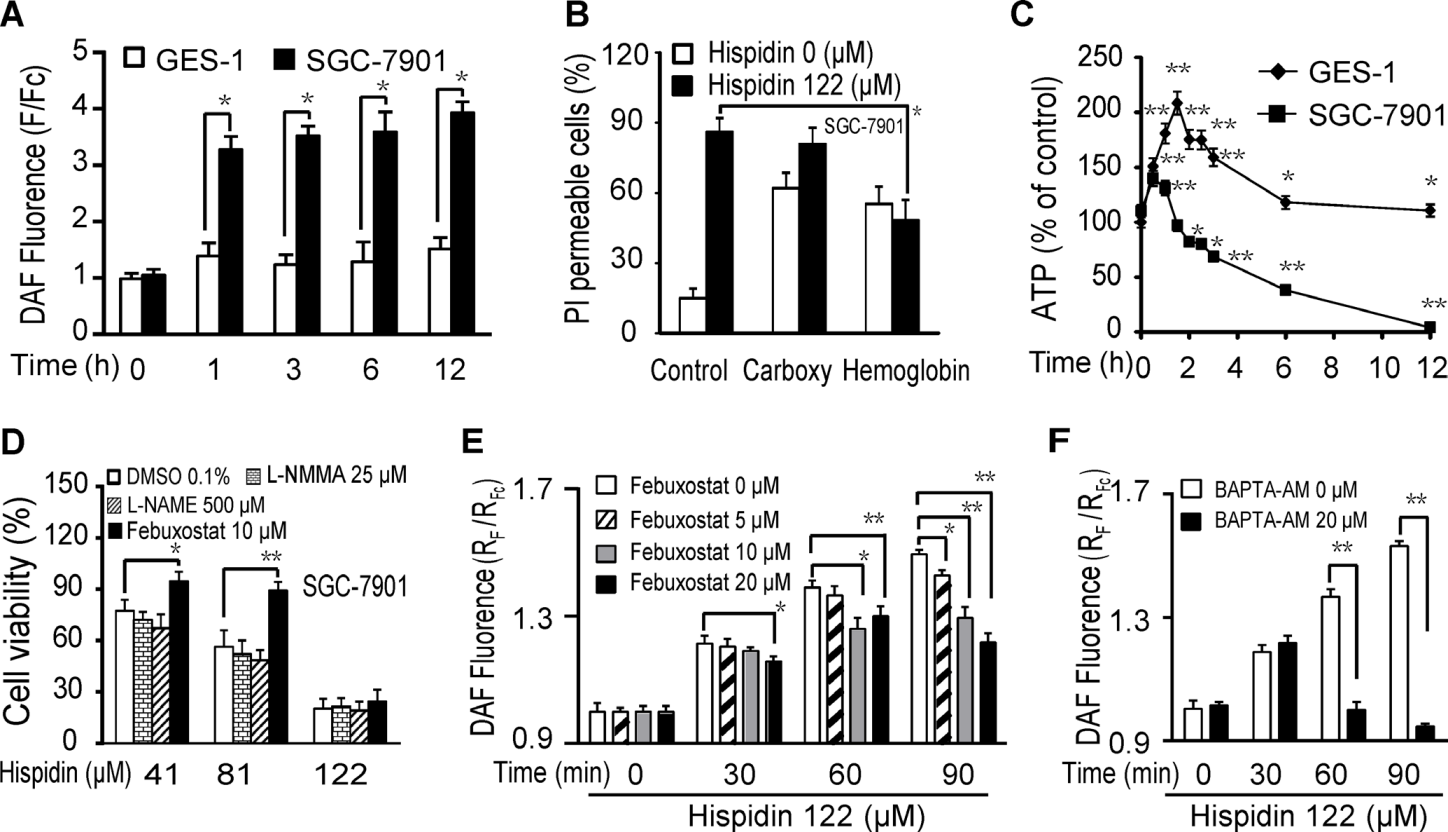
Hispidin induces LMP-related nitric oxide (NO) production, contributing to SGC-7901 cancer cell death (**A**) After being incubated with 122 μM hispidin or 0.1% DMSO for 0.5, 1, 2, or 3 h, SGC-7901 and GES-1 cells were assayed for DAF fluorescence. (**B**) After preincubation with either 20 μM carboxy-PTIO or 20 μM hemoglobin for 2 h, SGC-7901 cells were treated with 122 μM hispidin and assayed for PI permeability. (**C**) After being incubated with 122 μM hispidin or 0.1% DMSO, SGC-7901 and GES-1 cells were assayed for ATP. (**D**) After preincubation with L-NMMA, L-NAME and febuxostat for 2 h, SGC-7901 cells were treated with 122 μM hispidin or 0.1% DMSO for 24 h and assayed for cell viability using the MTT assay. (**E**) After preincubation with 0, 5, 10, 20 μM febuxostat for 2 h, SGC-7901 cells were stained with DAF-FM-DA, incubated with 122 μM hispidin, and assayed for DAF fluorescence (NO) for 90 min with a Microplate (**F**) After preincubation with 0, 20 μM BAPTA AM for 2 h, SGC-7901 cells were stained with DAF-FM-DA, incubated with 122 μM hispidin, and assayed for DAF fluorescence (NO) for 90 min with a Microplate Reader.

As shown in Figure 4A, 122 μM hispidin caused a 2.3-fold increase in NO (fluorescence of DAF) production in SGC-7901 cells after 1 h, but only a 0.3-fold increase was observed in GES-1 cells. Similar results were observed at 3, 6 and 12 h. The hispidin-induced increase in NO generation in SGC-7901 cells was also time- and concentration-dependent (Supplementary Figure 3). This indicates that hispidin exposure caused more NO production in SGC-7901 cancer cells than in GES-1 normal cells. Of the two NO scavengers used, 20 μM hemoglobin decreased the hispidin-induced PI permeability of SGC-7901 cells from 86% to 48%, but carboxyl-PTIO could not significantly reduce the hispidin-induced SGC-7901 cell death (Figure 4B). This indicates that hemoglobin prevented cell death through a function other than its NO scavenging activity. Rapid energy depletion did not occur after hispidin exposure (Figure 4C).

Generally, NO can be produced by NO synthase (NOS), xanthine oxidase and acidic reduction of nitrite in mammalian cells [11]. As shown in Figure 4D, neither the eNOS inhibitor L-NAME nor the total NOS inhibitor L-NMMA inhibited hispidin-induced SGC-7901 cell death. The xanthine oxidase inhibitor febuxostat significantly inhibited the increased production of NO and improved SGC-7901 cell viability (Figure 4D and 4E). Because NO generation through the reduction of nitrite by xanthine oxidase is an acid-catalyzed process [12], this result indicates that hispidin-induced acidic conditions related to LMP contributed to NO generation. As shown in Figure 4F, the intracellular calcium-specific chelator BAPTA-AM eliminated hispidin-induced NO generation, suggesting that free cytoplasmic Ca^2+^ from LMP may also promote the NO production. LMP-related destruction of redox systems increases oxidative stress in SGC-7901 cancer cells

**Figure 5:**
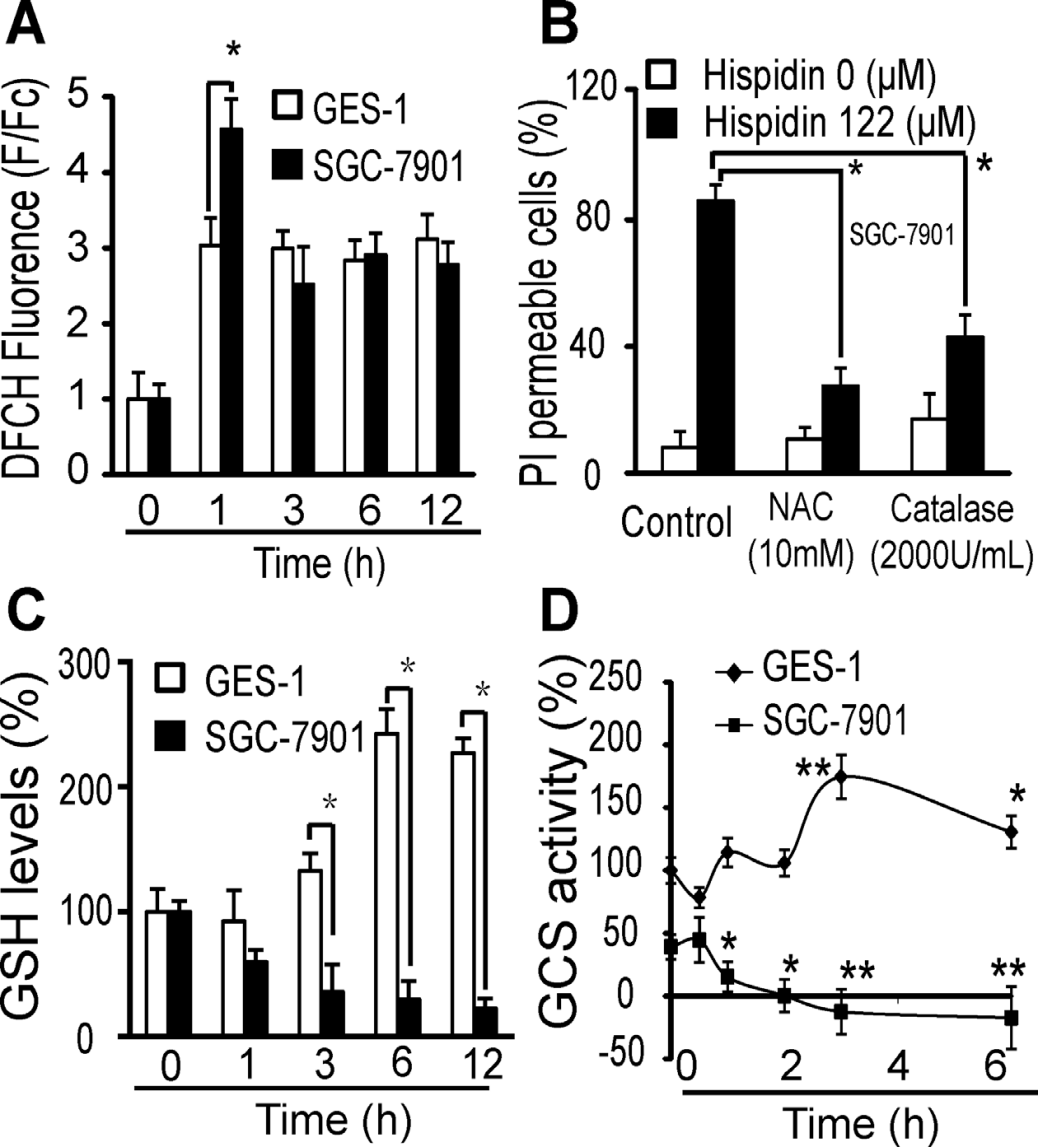
LMP-related redox system destruction increases oxidative stress in SGC-7901 cancer cells (**A**) After being incubated with 122 μM hispidin or 0.1% DMSO for 0.5, 1, 2, or 3 h, SGC-7901 and GES-1 cells were assayed for DCFH fluorescence. (**B**) After preincubation with either 2000 U catalase or 10 mM NAC for 2 h, SGC-7901 cells were treated with 122 μM hispidin and assayed for PI permeability. (**C**) After being incubated with 122 μM hispidin or 0.1% DMSO for 0.5, 1, 2, or 3 h, SGC-7901 and GES-1 cells were assayed for GSH. *P < 0.05 vs GES-1 samples treated under the same conditions. (**D**) SGC-7901 and GES-1 cells were incubated with 122 μM hispidin or 0.1% DMSO and assayed for GCS activity. The GCS activity of GES-1 cells at 0 h was used as the basic standard for both SGC-7901 and GES-1 cells.

After 1 h of treatment with 122 μM hispidin, the ROS levels (DCFH fluorescence) in SGC-7901 cells increased 4.6-fold, but they increased only 3-fold in GES-1 cells (Figure 5A). After 3 h, there was no significant difference in the fold increase in ROS levels between these two cell lines. Concentration-dependent ROS production was observed in SGC-7901 and GES-1 cells (Supplementary Figure 4A and 4B). Two antioxidants, 2000 U/mL catalase or 10 mM NAC, separately decreased the hispidin-induced PI permeability of SGC-7901 cells from 86% to 43% and 31%, respectively (Figure 5B), indicating that ROS significantly contributed to the hispidin-induced cell death. However, there was no loss of mitochondrial membrane potential (MMP) as early as six hours after hispidin treatment (Supplementary Figure 4C), showing that hispidin did not initiate cell death by directly disrupting the mitochondria [13].

The source of oxidative stress is not ROS generation per se, but the spatiotemporal imbalance in ROS reduction and detoxification. The GSH levels in GES-1 cells increased to 133%, 242% and 223% after 3, 6 and 12 h of treatment with 122 μM hispidin, respectively, but they decreased to 23% in SGC-7901 cells under the same conditions (Figure 5C). When different concentrations of hispidin were used, the GSH levels in GES-1 cells were significantly increased compared to those in SGC-7901 cells (Supplementary Figure 4D). We found that the γ-glutamylcysteine synthetase (γ-GCS) activity in untreated SGC-7901 cells was approximately 44% of that in untreated GES-1 cells. During treatment with 122 μM hispidin, the γ-GCS activity of GES-1 cells gradually increased to 170% at 3 h and then decreased to 131% at 6 h, but that of SGC-7901 cells decreased to zero within 2 h (Figure 5D). Hispidin did not inhibit the activity of purified *γ-GCS* or alter the expression of *γ-GCS* in either GES-1 or SGC-7901 cells (Supplementary Figure 4E and 4F). These results indicate that the destruction of the GSH redox system may be a secondary effect of hispidin-induced cellular destruction such as LMP. Thus, ROS would be more harmful to the GSH-depleted SGC-7901 cells.

### Hispidin induces microtubule depolymerization and is able to cause LMP

**Figure 6:**
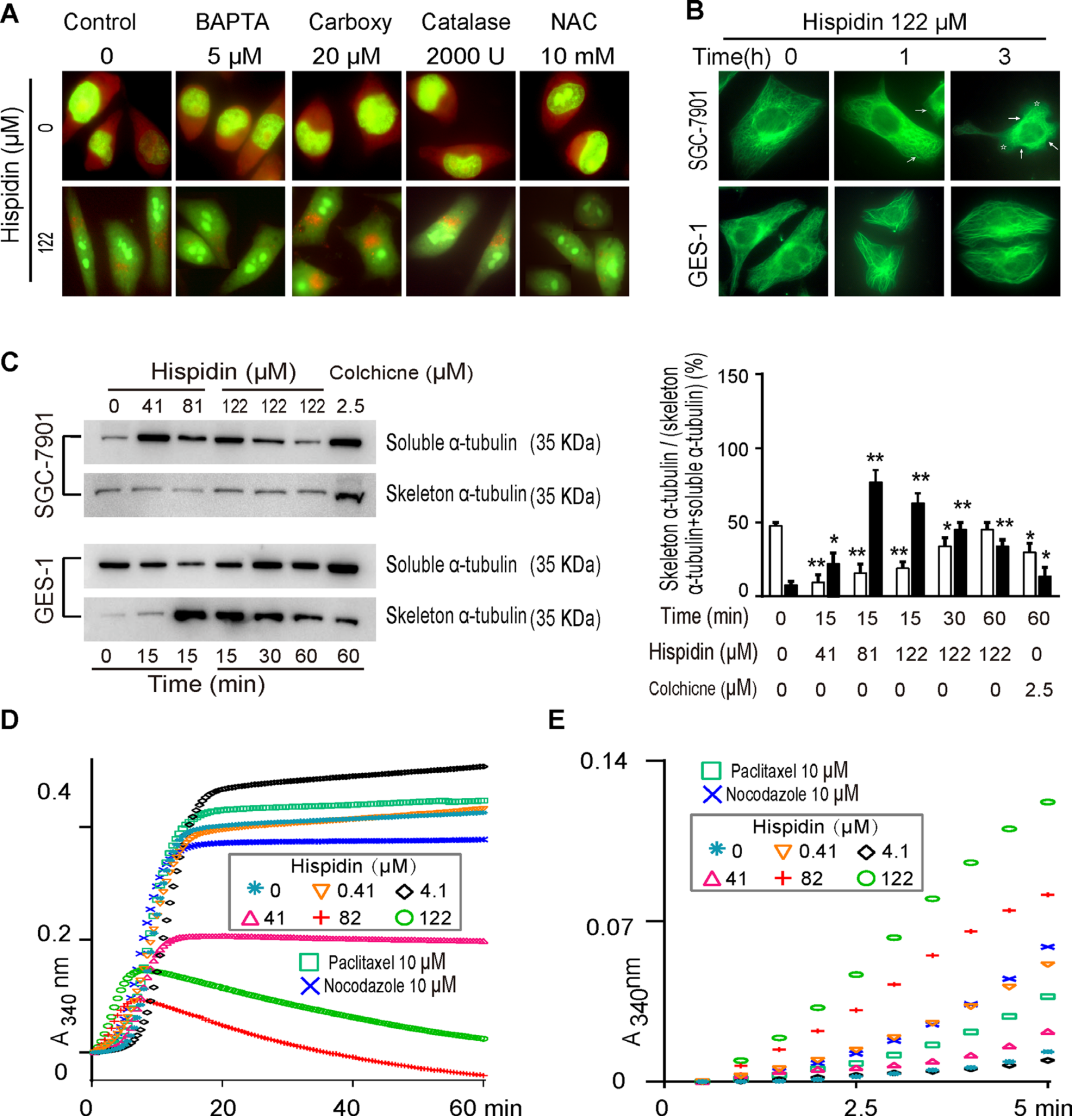
Hispidin induces microtubule depolymerization and is able to cause LMP (**A**) SGC-7901 cells were preincubated with 5 μM BAPTA AM, 20 μM carboxy PITO, 2000 U catalase or 10 mM NAC. Then, the cells were treated with 122 μM hispidin and stained with acridine Orange (AO). (**B**) SGC-7901 and GES-1 cells were incubated with 250 nM tubulin Tracker Green and 122 μM hispidin and assayed for fluorescence at different time points. (**C**) SGC-7901 and GES-1 cells were incubated with hispidin or 0.1% DMSO. Then, the α-tubulin in the cells was detected by Western blotting. (**D**) MAP-rich tubulin in a reaction buffer was incubated at 37°C in the presence of DMSO, hispidin, paclitaxel, and nocodazole. The polymerization of tubulin was determined by measuring the increase in absorbance over time at 340 nm. (**E**) Enlargement of Figure D in the early five min.

We further tracked the pathway of hispidin-induced LMP. First, the calpain–cathepsin pathway was excluded because an increase in cytoplasmic Ca^2+^ levels, which should have initiated the pathway, followed LMP in this study. This was also verified by the fact that BAPTA-AM could not efficiently prevent the LMP (Figure 6A). Next, neither antioxidants nor the lysosomotropic iron chelator desferrioxamine mesylate (DFO) could completely inhibit hispidin-induced LMP, indicating that the LMP was not initiated by ROS-mediated destabilization (Figure 6A). Furthermore, KIF5B, a member of the kinesin-1 family, was excluded because its depletion triggers peripheral lysosome aggregates, but hispidin induced perinuclear accumulation. In addition, endogenous cell death effectors such as Bax and p53 cannot induce such rapid LMP because their translocation exceeds the time required for the LMP here.

Because lysosomes move along the tubulin network, we studied the effect of hispidin on microtubule dynamics. Untreated SGC-7901 and GES-1 cells exhibited normal microtubule arrays, with filamentous microtubules radiating from the microtubule organizing center to the cell periphery (Figure 6B). After exposure to 122 μM hispidin, the microtubules promptly cracked or shortened to the area around the nucleus in SGC-7901 tumor cells. As a result, SGC-7901 cells shrank and often formed abnormal notches around their edges. However, there were no significant changes in the microtubules or shape of GES-1 cells. In Western blot analysis (Figure 6C), the α-tubulin skeleton of SGC-7901 cells dramatically decreased after 15 min of exposure to 122 μM hispidin and then slightly increased at 30 and 60 min. Under the same conditions, the α-tubulin skeleton of GES-1 cells decreased from the increased peak at 30 min and increased again at 60 min. At the end of the experiment, the α-tubulin skeleton of SGC-7901 cells was reduced compared to that at the beginning, while that in GES-1 cells was significantly increased. These results indicate that hispidin disrupts microtubule homeostasis.

To confirm that hispidin directly affects the organization of tubulin, a cell-free tubulin polymerization assay was conducted. As shown in Figure 6D, paclitaxel promoted the polymerization of tubulin into microtubules, whereas nocodazole inhibited it. No significant differences in tubulin polymerization were observed between samples without and with 0.4 μM hispidin; when used at 4.1 μM, hispidin significantly promoted tubulin polymerization. However, hispidin at 41, 81, and 122 μM decreased tubulin polymerization. Interestingly, although hispidin at 81 μM seemed to more drastically inhibit tubulin polymerization than 122 μM at 1 h, the increasing speed of tubulin polymerization at the early five min time was proportional to the hispidin concentration (Figure 6E).

### Stathmin 1 (STMN1) phosphorylation and dephosphorylation is involved in hispidin-induced microtubule depolymerization

**Figure 7:**
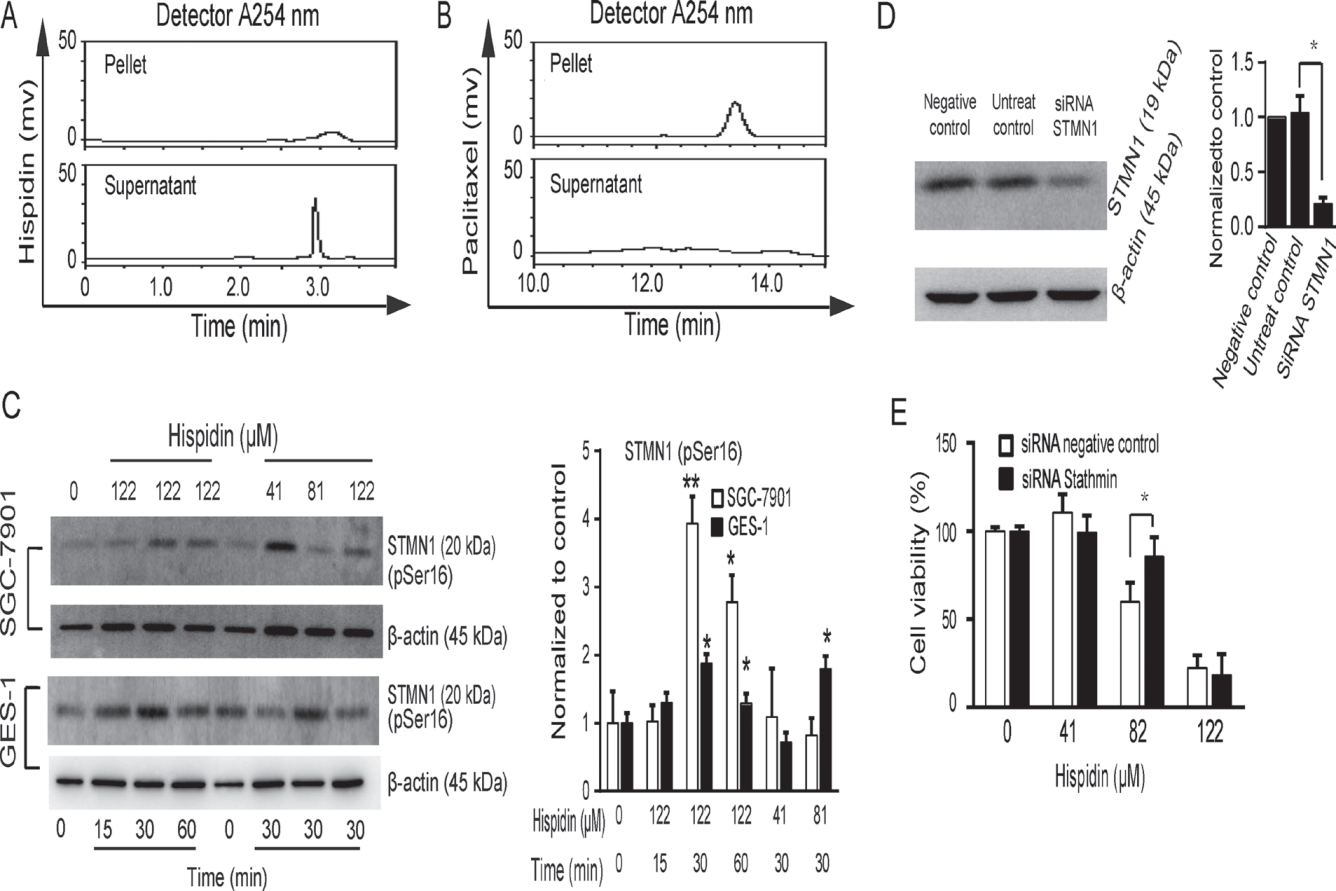
STMN1 phosphorylation and dephosphorylation is involved in hispidin-induced microtubule depolymerization (**A** and **B**) Tubulin was purified, cross-linked and used as a receptor in hispidin and paclitaxel binding assays. (**C**) SGC-7901 and GES-1 cells were incubated with hispidin or 0.1% DMSO. Then, STMN1 phosphorylation at ser^16^ was detected by Western blotting using β-actin as an internal control. (**D**) SGC-7901 cells were transfected with scrambled RNA and STMN1 RNA for 48 h, and STMN1 levels were determined by Western blot. (**E**) The transfected cells were treated with 122 μM hispidin and assayed for viability.

Drugs can modulate microtubule dynamics by binding to microtubules or affecting microtubule regulatory proteins. As shown in Figure 7A, hispidin was detected in the supernatant without microtubules, but paclitaxel was detected in the pelleted extracts of the cross-linked microtubules (Figure 7B), indicating that hispidin did not disrupt microtubule stability by binding.

Next, we tried to identify some key microtubule regulatory proteins involved in hispidin-induced cell death, such as the kinesin-13 family and STMN1/oncoprotein18. STMN1 is necessary for entry into prophase and termination in cytokinesis, while the kinesin-13 family plays major roles in spindle dynamics and accurate chromosome segregation [14]. The catastrophe-promoting activity of STMN1 can be inactivated by the phosphorylation of either Ser^16^ or Ser^63^. The Ser^16^ site is conserved in the STMN1 family. Thus, we examined the phosphorylation of Ser^16^ after hispidin treatment. STMN1 phosphorylation was increased at 30 min and then decreased again at 60 min in SGC-7901 cells treated with 122 μM hispidin (Figure 7C). However, only very slight changes in STMN1 phosphorylation were observed in GES-1 cells under the same conditions. Thus, the hispidin-induced reversible phosphorylation of STMN1 may be one reason for the disrupted microtubule stability. To identify the role of STMN1 in hispidin-induced cell death, we conducted RNA interference (Figure 7D). As shown in Figure 7E, STMN1 siRNA attenuated SGC-7901 cell death only when the concentration of hispidin applied was lower than 81 μM. This indicates that hispidin may affect different microtubule regulatory proteins with the variation of its concentration.

### Hispidin induces similar death in A549 and HepG2 cancer cells as in SGC-7901 cells but no death in L02 normal cells

To explore whether hispidin can cause cell death in other cancer cells as it did in SGC-7901 cells, we treated A549 and HepG2 cancer cells and L02 normal cells with hispidin and examined cell viability (Supplementary Figure 5A to 5C), cell death (Supplementary Figure 5D), morphological changes (Supplementary Figure 5E), LMP (Supplementary Figure 5F) and tubulin depolymerization (Supplementary Figure 5G). Hispidin caused similar cell death in A549 and HepG2 cells as it did in SGC-7901 cells. Consistently, there was still no significant death in L02 cells as in GES-1 cells.

## DISCUSSION

The induction of autophagic or necrotic cell death is an alternative approach to eliminate cancer cells [15–18]. This study shows that hispidin induced necrotic cell death involving autophagy in SGC-7901 cancer cells by reversibly phosphorylating microtubule regulatory proteins, such as STMN1, disrupting microtubule homeostasis and inducing LMP. By contrast, hispidin exhibited nearly no cytotoxicity against GES-1 gastric cells under the same conditions. Efficient utilization of the cellular differences between normal and cancer cells may contribute to the higher cytotoxic activity of hispidin against SGC-7901 cells.

Microtubule regulatory proteins such as STMN1 may play critical roles in hispidin-induced cell death. STMN1 is a novel and important oncogene [19–21]. Dephosphorylated STMN1 could bind to two free tubulin-GDP dimers and form a stable complex that cannot polymerize into microtubules or directly bind to microtubules and increases the catastrophe frequency [22]. Remarkably, the hispidin-induced STMN1 phosphorylation at Ser^16^ can be rapidly reversed and restored. This most likely resulted from the down- and up-regulation of the activity of at least two kinases, such as p21-activated kinase 1 (PAK1), Aurora B, CaMKII, and IV/G. In accord with this finding, hispidin strongly inhibited PAK1 (IC50 = 5.7 μM) [23–25], which promoted STMN1 dephosphorylation at Ser^16^. By contrast, LMP caused by microtubule depolymerization increased the free cytoplasmic calcium concentration, which up-regulated the activity of CaMKII and some other calcium-dependent kinases to enhance STMN1 phosphorylation at Ser^16^. In addition, Ser^63^ could be simultaneously phosphorylated by PKA and the binding affinity of STMN1 for tubulin heterodimers could be reduced. Moreover, the phosphorylation of Ser^25^ and Ser^38^ by Cdk5, MAPK, MAPKd and Cdk1/cdc2 could regulate the phosphorylation status of Ser^16^ and Ser^63^ but have little apparent effect on tubulin binding [26]. Notably, hispidin could induce larger fluctuations in microtubule regulatory proteins, such as STMN1 phosphorylation at Ser^16^, in SGC-7901 cells than in GES-1 cells because cancer cells usually exhibit higher expression levels or activity of these kinases.

LMP is one mechanism for the induction of cell death [27–30]. Notably, compared to normal cells, cancer cells contain larger and more fragile lysosomes that are redistributed from a perinuclear to a peripheral pattern [31]. When microtubules fluctuate, LMP tends to occur in peripheral lysosomes, which constitute the majority of the lysosomes in cancer cells but a minority of the lysosomes in normal cells. Furthermore, cancer cells display higher metabolic rates and increased turnover of iron-containing proteins, leading to the lysosomal accumulation of iron [32]. Even if hispidin induces ROS similarly in cancer and normal cells, it could generate more oxygen radicals in cancer cells through Fenton-type reactions using their higher intra-lysosomal iron content, thus helping to trigger LMP. In addition, cysteine cathepsins are highly up-regulated and mainly stored in the lysosomes of various cancers [33]. When hispidin-induced LMP occurred in SGC-7901 cells, more cathepsins were released to the cytoplasm, which in turn significantly aggravated the LMP.

In summary, hispidin induces the autophagic and necrotic death of SGC-7901 gastric cancer cells via LMP through inhibiting tubulin polymerization by modulating phosphorylation of microtubule regulatory proteins such as STMN1. The underlying molecular mechanism suggests a promising novel treatment for human cancer.

## MATERIALS AND METHODS

### Reagents and antibodies

Hispidin (purity > 99%) was synthesized as previously reported [34]. RPMI 1640, DMEM, FBS and 0.25% trypsin were purchased from Thermo Fisher Scientific (Shanghai, China). MTT, necrostatin-1, hemoglobin, N-acetyl-L-cysteine (NAC), Catalase, Carboxy-PITO, the TUNEL kit, Hoechst 33342, rapamycin, 3-Methyladenine, wortmannin, bafilomycin A1, hydroxychloroquine, E64d and pepstatin A were purchased from Sigma-Aldrich (St. Louis, MO, USA). Cathepsin inhibitor 1 was purchased from AbMole BioScience (Shanghai, China). The broad-spectrum caspase inhibitor z-VAD-fmk was purchased from BD Biosciences (San Jose, CA, USA). The mCherry-GFP-LC3B reporter was purchased from Beyotime Biotechnology (Jiangsu, China). The mouse anti-caspase-8 monoclonal antibody, rabbit monoclonal anti-caspase-3, anti-β-actin, anti-caspase-9, anti-LC3A/B and anti-STMN1 phosphorylated ser^16^ antibodies were from Cell Signaling Technology (Beverly, MA, USA). The anti-γ-GCS, horseradish peroxidase-conjugated anti-rabbit and anti-mouse secondary IgG antibodies were from Santa Cruz Biotechnology (Santa Cruz, CA, USA). STMN1 siRNA SMART pools were purchased from Dharmacon (Lafayette, CO, USA). Lipofectamine 2000 was purchased from Invitrogen (Carlsbad, CA, USA). A tubulin polymerization assay kit was purchased from Cytoskeleton (Denver, CO, USA)

### Cell cultures and cell viability assay

The SGC-7901 human gastric cancer cell line and GES-1 immortalized human gastric epithelial cell line were obtained from the Institute of Biochemistry and Cell Biology, Chinese Academy of Sciences. The SGC-7901 cells were cultured in RPMI 1640 containing 10% FBS at 37°C in a 95% air and 5% CO_2_ humidified incubator; GES-1 cells were cultured in DMEM containing 10% FBS under similar conditions. Hispidin was dissolved in dimethyl sulfoxide (DMSO) at a concentration of 100 mg/mL and diluted to the required concentration before use. Unless otherwise indicated, Adriamycin was used as a positive control, and DMSO (0.1%) was used as a negative control.

Cell viability was determined using the MTT assay. The cells were seeded at a density of 3 × 10^3^ cells per well in 96-well microtiter culture plates, incubated overnight, and treated with various concentrations of hispidin for 24, 48, and 72 h. Then, the cells were washed three times with PBS, incubated with 0.5 mg/mL of MTT for 4 h at 37°C, lysed with DMSO and measured in a scanning multi-well spectrophotometer at 490 nm. Apoptotic rates were analyzed by flow cytometry using the Annexin V-FITC/PI kit according to the manufacturer's instructions. Alternatively, apoptotic cells were visually examined using Hoechst 33342 staining and the TUNEL kit (BD Biosciences). Caspase-3, caspase-8 and caspase-9 activities were analyzed using the Apo-ONE Homogenous Caspase-3/8/9 assay kit (Promega).

### Cell cycle analysis

The cells were trypsinized, washed twice, resuspended with PBS, and then cold absolute ethanol was added to a concentration of 70%. After the cells were fixed on ice for at least 30 min, 1 × 10^6^ cells were incubated with 0.1 mg/mL RNase A in PBS for 30 min at 37°C. The samples were treated with 5 μg/mL propidium iodide and analyzed with a BD LSR II flow cytometer (BD Biosciences, San Jose, CA, USA).

### Autophagic flux assay

SGC-7901 cells were transfected with an adenovirus expressing the dual fusion protein mCherry-GFP-LC3B according to the manufacturer's instructions. Autophagic flux was observed as reported previously [35].

### Electron microscopy

The cells were trypsinized, washed, and fixed in 2.5% glutaraldehyde for 30 min. The samples were treated with 1% osmium tetroxide, dehydrated with acetone, and embedded in Durcupan resin. Thin sections collected on grids were stained with uranyl acetate and lead citrate and then examined with an H-7650 transmission electron microscope.

### Measurement of the mitochondrial membrane potential and GSH/GSSG

The mitochondrial membrane potential (MMP) was measured with the JC-1 mitochondrial membrane potential assay kit (Cayman) by flow cytometry according to the manufacturer's instructions. GSH/GSSG was measured with a kit from BioVision. The GSH/GSSG concentrations were normalized to the protein content of the lysates.

### Measurement of free cytosolic Ca^2+^, ROS and nitrogen oxide (NO) levels

The cells were trypsinized, washed, and loaded with 2 μM Fluo-3 AM (for Ca^2+^ measurements) in standard Tyrode's solution (135 mM NaCl, 4 mM KCl, 1 mM MgCl_2_, 2 mM CaCl_2_, 10 mM glucose, and 5.0 mM HEPES, pH 7.3), 1 μM DCFH-DA (for ROS measurements) in phenol-free growth medium, or 0.5 μM DAF-FM-DA (for NO measurements) in phosphate buffer (0.1 M phosphate, pH 7.4). The cells were then washed, resuspended, and analyzed by flow cytometry. The variations in Ca^2+^, ROS and NO concentration were expressed as F/F_c_, *i.e*., the drug-induced fluorescence (F) relative to the fluorescence of the control (F_c_).

Alternatively, the intracellular concentrations of Ca^2+^, ROS and NO were measured using the Tecan Infinite M200 Microplate Reader. Briefly, the cells were seeded in 96-well black opaque plates (Corning) at a density of 3 × 10^3^ cells per well in phenol red-free growth medium. After an overnight incubation, the cells were washed and loaded with Fluo-3 AM, DCFH-DA or DAF-FM-DA for 30 min at 37°C. Then, the cells were washed twice, supplemented with phenol red-free growth medium with or without drugs, and the fluorescence was immediately measured every 30 s for 90 min at 37°C. The variations in Ca^2+^, ROS and NO concentration were expressed as R_F_/R_Fc_. R_F_ represents the ratio of the fluorescence of the experimental group at the tested time to that at the starting time point, and R_Fc_ is the fluorescence of the control group (0.1% DMSO).

Calcium (Fluorescence of Fluo-3 AM) was colocalized with the endoplasmic reticulum (Fluorescence of ER Tracker Red), lysosomes (Fluorescence of LysoTracker Red) or mitochondria (Fluorescence of MitoTracker Red) using confocal laser scanning microscope (Zeiss, Oberkochen, Germany).

### Sarcoendoplasmic reticulum Ca^2+^-ATPase (SERCA) and γ-glutamylcysteine synthetase (γ-GCS) activity assays

SERCA and γ-GCS activities were detected as previously described [36, 37].

### Detection of lysosomal membrane permeabilization

Lysosomal membrane permeabilization (LMP) was measured with LysoTracker red and acridine orange (AO). Cells grown in phenol red-free growth medium were stained with 41 μM acridine orange for 15 min or 50 nM LysoTracker red for 30 min according to the manufacturer's instructions. Then, the cells were rinsed and examined using a fluorescence microscope.

### Live cell labeling with a tubulin tracker

The cells were washed with Kreb's buffer containing 2 mM Ca^2+^, incubated with 250 nM of tubulin Tracker Green Reagent (Invitrogen) for 30 min at 37°C, rinsed 3 times with Kreb's buffer containing 2 mM Ca^2+^, and viewed under a fluorescence microscope.

### Extraction of solubilized and polymerized tubulin

After exposure to hispidin or the positive control colchicine, the culture medium was removed. Cells were washed with PBS; solubilized and polymerized (cytoskeletal) tubulin fractions were collected as described previously [38].

### Microtubule polymerization analysis

Microtubule polymerization assays were performed using a fluorescence-based tubulin polymerization assay kit (Cytoskeleton, Denver, CO, USA) according to the manufacturer's protocol.

### Microtubule binding assay

Tubulin was prepared from calf brain and assembled and cross-linked into microtubules as previously described [39]. The microtubule binding assay was conducted as previously described, with slight modifications [40]. Hispidin and paclitaxel were separately incubated with the cross-linked microtubules in glycerol assembly buffer (3.4 M glycerol, 10 mM sodium phosphate, 1 mM EGTA, 6 mM MgCl_2_, and pH 6.5) with 0.1 mM GTP for 30 min at 25°C. The samples were then centrifuged at 90,000 × g for 15 min at 25°C using a Beckman Optima TLX ultracentrifuge. The supernatant was collected, and the pellet was resuspended in 10 mM phosphate buffer (pH 7.0). Both the pellet and supernatant were extracted three times with an excess volume of dichloromethane, dried under vacuum, and dissolved in methanol. The amount of hispidin or paclitaxel that was bound to the pelleted polymers or present in the supernatant was determined by HPLC using a C-18 column (Shim-pack VP-ODS, 250 × 4.6 mm, 5 mm bead size) with 70% methanol in water (v/v) at a flow rate of 1 mL/min.

### Western blot analysis

The cells were treated with the indicated concentration of hispidin for various times. The total protein (μg) was applied to a 10–12% SDS-PAGE gel, transferred onto a nitrocellulose membrane, detected with the appropriate primary and secondary antibodies and then visualized with a chemiluminescence kit (Pierce).

### siRNA silencing

siRNAs against human *STMN1* were purchased form Dharmacon (DDIT smart pool siRNA). siRNAs against human ATG5 were bought from Qiagen (Valencia, CA, USA). SiRNAs were transfected into cells using Lipofectamine 2000 (Invitrogen) as described previously (33).

### Statistical analysis

Each experiment was performed at least three times to ascertain the reproducibility of the result. Student's *t* test was used to determine the statistical significance. The data were expressed as the means ± s.e.m. All error bars were based on the s.e.m. *P <* 0.05 was considered as significant. Asterisks denote *P < 0.05, **P < 0.01.

## SUPPLEMENTARY MATERIALS FIGURES AND TABLES


