# Supplementary material for: Hispidin induces autophagic and necrotic death in SGC-7901 gastric cancer cells through lysosomal membrane permeabilization by inhibiting tubulin polymerization
Source: Oncotarget. 2017 Mar 6;8(16):26992–7006. doi: 10.18632/oncotarget.15935 (PMC5432313; doi:10.18632/oncotarget.15935)
Supplement: Supplementary file 1 [file oncotarget-08-26992-s001.pdf]

Hispidin induces autophagic and necrotic death in SGC-7901 gastric cancer cells through lysosomal membrane permeabilization by inhibiting tubulin polymerization

Supplementary Materials

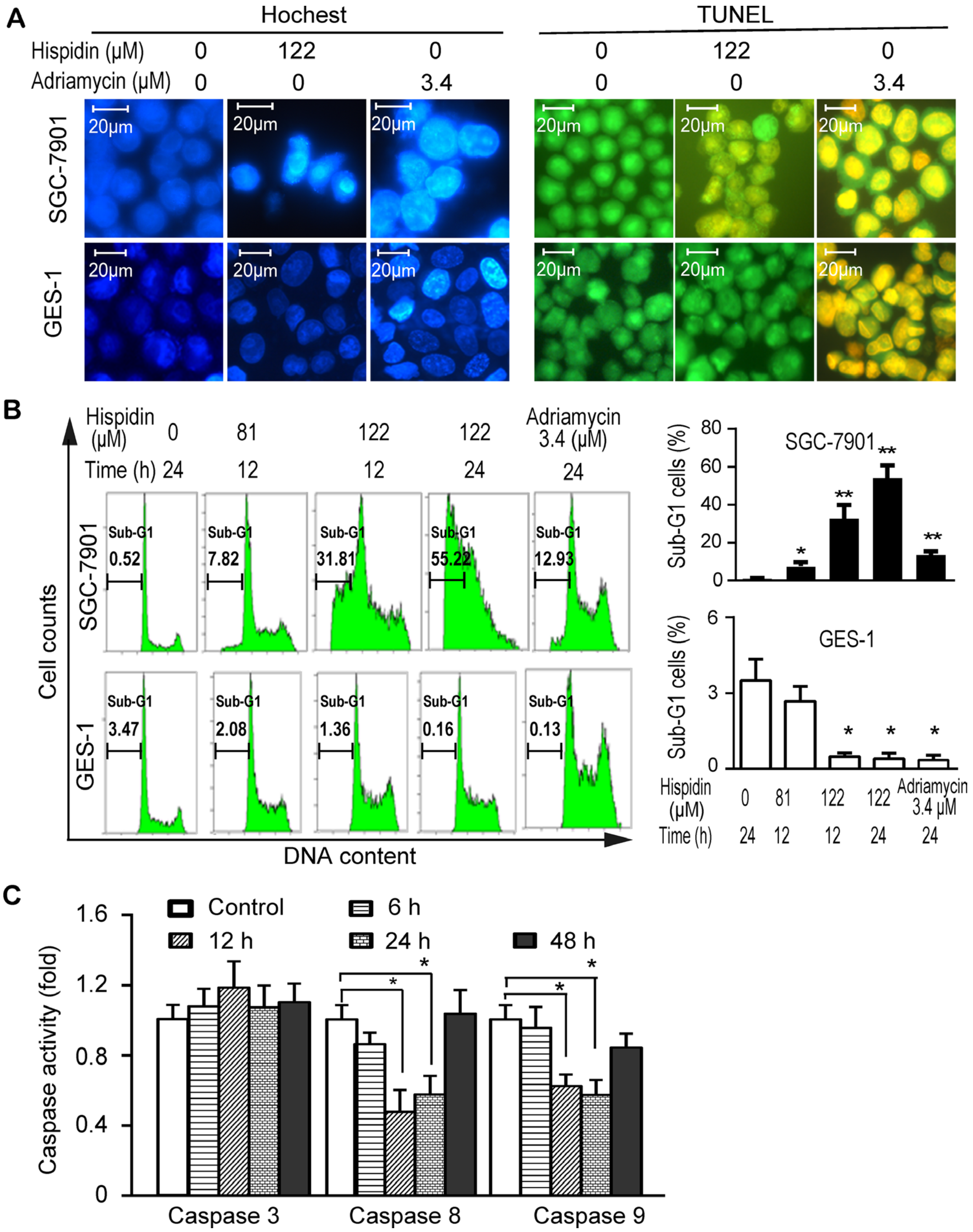

Supplementary Figure 1: Hispidin induces caspase-independent cell death in SGC-7901 cells. (**A**) Cells were treated with hispidin, 0.1% DMSO, or Adriamycin for 24 h. Then, the cells were stained with Hoechst 33342 or the TUNEL kit. (**B**) Cells were incubated with hispidin or 0.1% DMSO, and the cell cycle was assayed. (**C**) SGC-7901 cells were treated with 122 μM hispidin, 0.1% DMSO, or 3.4 μM Adriamycin for 6 h, 12 h, 24 h and 48 h. Then, their caspase activity was examined.

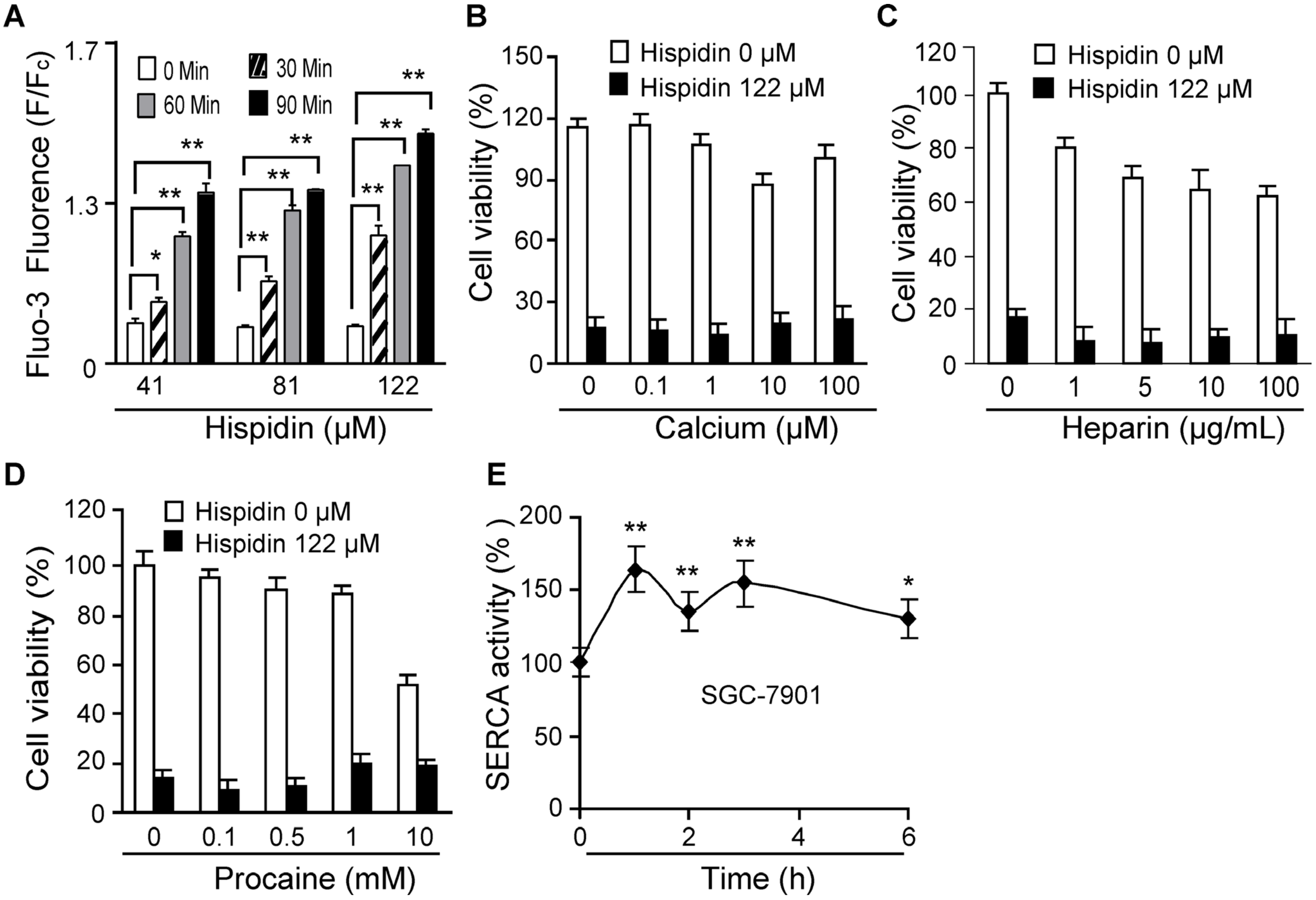

Supplementary Figure 2: Hispidin induces lysosomal membrane permeabilization (LMP). (**A**) Hispidin increased the cytoplasmic Ca^{2+} concentration in SGC-7901 and GES-1 cells. After staining with Fluo-3 AM, cells were incubated with hispidin and assayed for fluorescence for 90 min using a Microplate Reader. (**B**) SGC-7901 cells were treated with 122 mM hispidin or 0.1% DMSO in growth medium with different concentrations of calcium for 24 h and assayed for cell viability using the MTT assay. SGC-7901 cells were preincubated with heparin (1, 5, 10, or 100 μg/mL) (**C**) or procaine (0.1, 0.5, 1, or 10 mM) (**D**) for 2 h. Then, the cells were treated with 122 μM hispidin or 0.1% DMSO for 24 h, and cell viability was measured using the MTT assay. (**E**) SGC-7901 cells were incubated with 122 μM hispidin or 0.1% DMSO and then were assayed for SERCA activity.

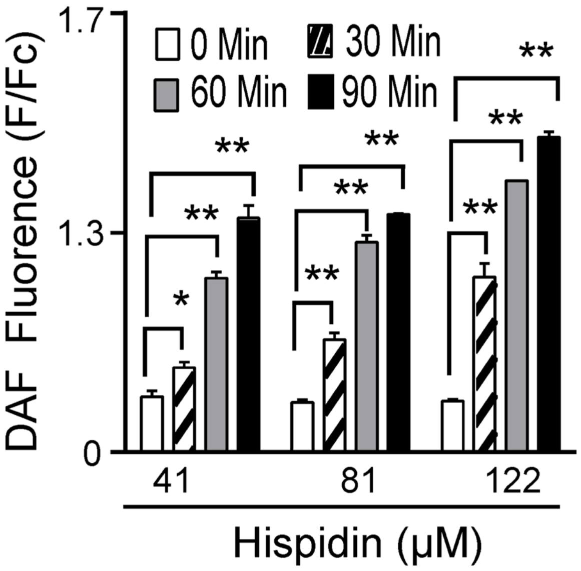

Supplementary Figure 3: The hispidin-induced increase in NO generation in SGC-7901 cells was time- and concentration-dependent. After staining with DAF-FM-DA, cells were incubated with hispidin and assayed for fluorescence for 90 min using a Microplate Reader.

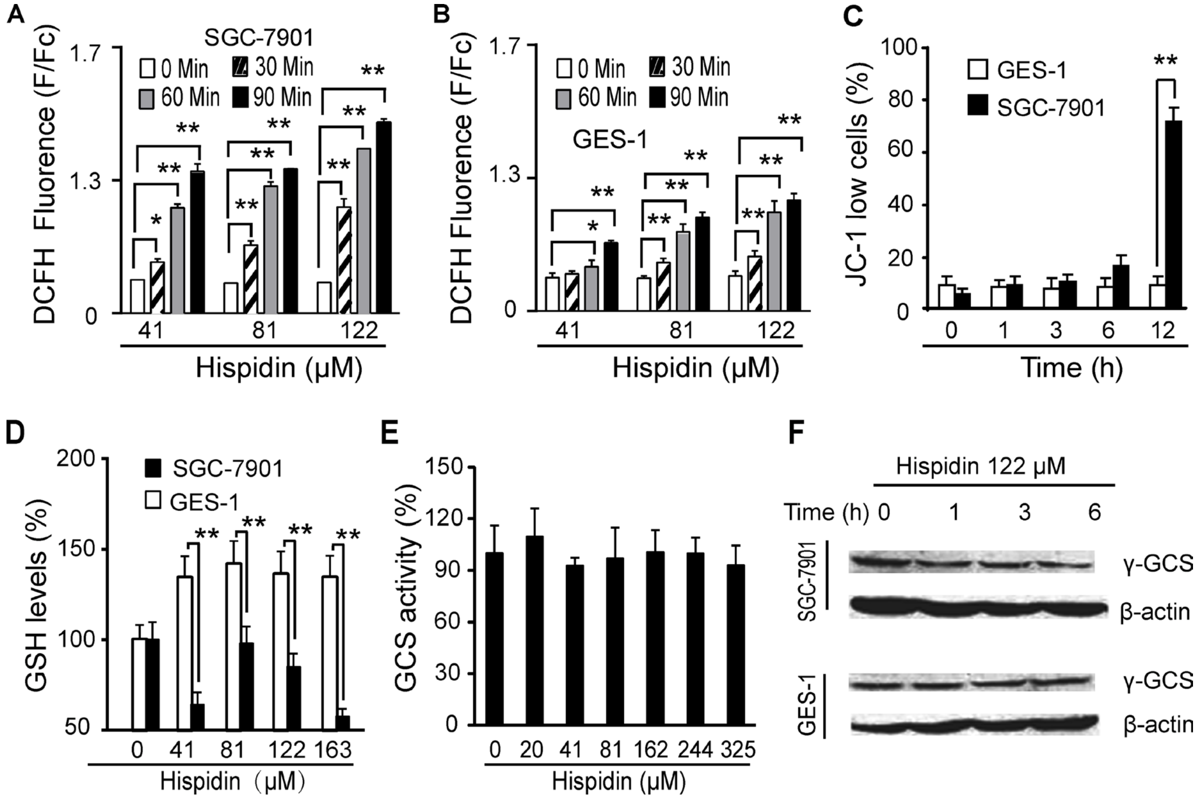

Supplementary Figure 4: LMP-related redox system destruction increases oxidative stress in SGC-7901 cancer cells. After staining with DCFH-DA, SGC-7901 cells (**A**) and GES-1 cells (**B**) were incubated with hispidin and assayed for fluorescence for 90 min using a Microplate Reader. (**C**) After being incubated with 122 μM hispidin or 0.1% DMSO for 0.5, 1, 2, 3 h, the SGC-7901 and GES-1 cells were assayed for JC-1 low cells. (**D**) After being incubated with different concentrations of hispidin for 3 h, GSH levels in SGC-7901 and GES-1 cells were assayed. (**E**) Purified GCS was incubated with 20 to 325 μM hispidin and assayed for GCS activity. (**F**) SGC-7901 and GES-1 cells were incubated with 122 μM hispidin or 0.1% DMSO for 1, 3, and 6 h, and then γ-GCS was detected by Western blotting using β-actin as an internal control.

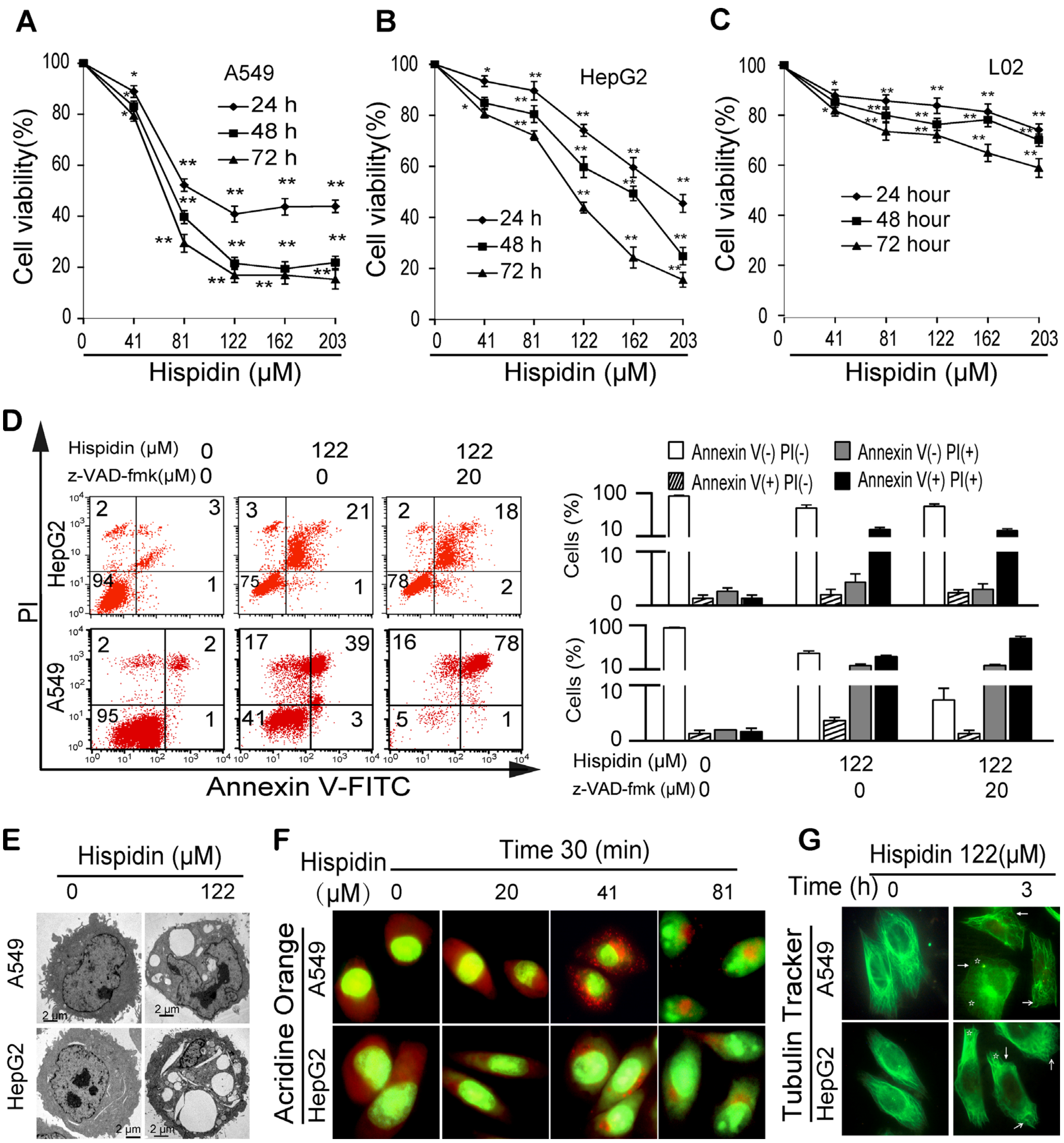

Supplementary Figure 5: Hispidin induces similar death in A549 and HepG2 cancer cells as in SGC-7901 cells but no death in L02 normal cells. A549 (**A**), HepG2 (**B**) and L02 (**C**) cells were incubated with hispidin (41, 81 or 122 μM) or 0.1% DMSO for 24, 48 or 72 h. The viability of cells was determined using the MTT assay. A549 and HepG2 (**D**) cells were preincubated with or without 20 μM z-VAD-fmk. Then, the cells were incubated with hispidin for 24 h and assayed for phosphatidyl serine externalization and PI permeability. (**E**) Transmission electron microscopy of the cancer cells. (**F**) Cells were treated with 20, 41, or 81 μM hispidin for 30 min and then were tested for lysosomal membrane permeabilization with acridine orange. (**G**) Cells were incubated with 250 nM tubulin Tracker Green and 122 μM hispidin and assayed for fluorescence at 0 and 3 h.
